# Structure-Based Virtual Screening of Potential Inhibitors Targeting the Prolyl-tRNA Synthetase (PRS) in *Eimeria tenella*: Insights from Molecular Docking, ADMET Studies, and Molecular Dynamics Simulations

**DOI:** 10.3390/molecules30040790

**Published:** 2025-02-08

**Authors:** Haiming Cai, Shenquan Liao, Juan Li, Minna Lv, Xuhui Lin, Yongle Song, Xiangjie Chen, Yibin Zhu, Jianfei Zhang, Nanshan Qi, Mingfei Sun

**Affiliations:** Key Laboratory of Livestock Disease Prevention of Guangdong Province, Key Laboratory of Avian Influenza and Other Major Poultry Diseases Prevention and Control, Ministry of Agriculture and Rural Affairs, Institute of Animal Health, Guangdong Academy of Agricultural Sciences, Guangzhou 510640, China; caihaiming@gdaas.cn (H.C.); liaoshenquan@gdaas.cn (S.L.); lijuan@gdaas.cn (J.L.); lvminna@gdaas.cn (M.L.); linxuhui@gdaas.cn (X.L.); songyongle@gdaas.cn (Y.S.); chenxiangjie@gdaas.cn (X.C.); zhuyibin@gdaas.cn (Y.Z.); zhangjfei@tom.com (J.Z.)

**Keywords:** avian coccidiosis, prolyl-tRNA synthetase, *Eimeria tenella*, docking protocol, molecular dynamics simulations, ADMET

## Abstract

Avian coccidiosis, caused by protozoan parasites of the genus *Eimeria*, poses a major threat to the poultry industry worldwide, leading to severe economic losses through reduced growth rates, poor feed efficiency, and increased mortality. Although the conventional management of this disease has relied on anticoccidial drugs, the overwhelming use of these agents has led to the rapid emergence and spread of drug-resistant *Eimeria* isolates, highlighting the urgent need for novel therapeutic approaches. This study employed computational approaches to identify novel inhibitors targeting *Eimeria tenella* prolyl-tRNA synthetase (EtPRS). Based on the virtual screening of a library of 3045 natural compounds, 42 high-confidence inhibitors were identified. Three compounds, including Chelidonine, Bicuculline, and Guggulsterone, demonstrated strong and selective binding to EtPRS through stable interactions within the active site. ADMET predictions revealed favorable safety profiles, while molecular dynamic simulations confirmed binding stability. Overall, this research established a solid framework for the development of effective anticoccidial agents targeting PRS, contributing to the advancement of therapeutic strategies for combating parasitic infections in the poultry industry.

## 1. Introduction

Chicken coccidiosis, a parasitic disease caused by protozoa of the *Eimeria* genus, poses a significant challenge to the global poultry industry. This disease inflicts heavy economic losses, estimated at around USD 3 billion annually, primarily due to decreased growth rates, impaired feed efficiency, and elevated mortality in infected flocks [1,2]. Apart from these financial implications, coccidiosis adversely affects food security and animal welfare on a global scale. Historically, the treatment of coccidiosis has relied heavily on anticoccidial drugs, including polyether antibiotics (such as monensin and salinomycin) and synthetic compounds (such as amprolium and diclazuril) [3,4,5]. These drugs have played a key role in containing the disease and ensuring productivity in the poultry industry. Nevertheless, the often excessive use of anticoccidial agents has led to the rapid emergence and spread of drug-resistant *Eimeria* strains [6,7]. Consequently, this phenomenon has severely undermined the effectiveness of existing treatments, highlighting the urgent need to formulate novel therapeutic strategies.

Halofuginone (HFG), a halogenated product derived from the natural compound febrifugine, has shown outstanding effectiveness among the anticoccidial agents against apicomplexan parasites, including *Eimeria* spp. [8,9]. Its high efficacy has made HFG a valuable tool in combating coccidiosis. Moreover, recent research has revealed crucial insights into its mechanism of action, paving new pathways for advanced drug development and resistance management. The identification of prolyl-tRNA synthetase (PRS) as a molecular target of HFG in *Plasmodium falciparum*, another apicomplexan parasite, marks a breakthrough in elucidating its mode of action [10,11]. PRS, a vital member of the aminoacyl-tRNA synthetase (AARS) family, is essential for protein synthesis as it catalyzes the covalent attachment of proline to its corresponding transfer RNA (tRNA) [12]. This process is vital for accurate genetic translation and parasite survival. Its high sequence conservation among apicomplexan parasites also underscores the significance of PRS as a potent drug target [13] and its potential use as a reliable molecular marker for early detection of HFG resistance in *Eimeria* spp., along with the management of resistant strains [14]. Additionally, the conserved nature of PRS across species offers a promising approach for the development of broad-spectrum antiparasitic agents.

Structural studies have revealed that PRS consists of two key domains: a catalytic domain (CD) and an insertion (INS) domain [15]. The former is directly involved in the aminoacylation reaction, while the latter is essential in substrate binding and activation [16]. The active site of PRS encompasses three distinct pockets that bind ATP, proline, and the 3′-terminal adenosine residue of tRNAPro (A76), respectively [17], thereby providing numerous potential binding sites for inhibitors. Furthermore, HFG has been recognized as an effective PRS inhibitor, attributed to its distinctive structural properties that allow it to mimic both adenine and L-proline, thus enabling dual-site inhibition of PRS [18]. This dual-targeting mechanism enhances HFG’s potency and minimizes the risk of resistance arising through single-point mutations. Despite these advantages, emerging HFG resistance highlights the need to formulate new inhibitors that target the same or alternative active sites on PRS.

Computational approaches provide substantial benefits in the initial stages of drug discovery, facilitating rapid large-scale screening of compound libraries and elucidating protein–ligand interactions. Molecular docking is one of the vital computational techniques that enable the virtual screening of millions of compounds against a target protein structure, which identifies potential inhibitors according to their predicted binding affinities and interactions [19]. Complementing this, molecular dynamics (MD) simulations offer a dynamic perspective on protein–ligand complexes, revealing details about the conformational changes, binding stability, and potential allosteric effects that static structures fail to recognize [20]. These structure-based simulations yield crucial insights into the mechanism of action of potential inhibitors and help anticipate their efficacy and mode of resistance. In addition, the incorporation of ADMET (Absorption, Distribution, Metabolism, Excretion, and Toxicity) predictions into drug discovery research has become increasingly vital [21]. In particular, ADMET analysis evaluates the drug-likeness and toxicity profiles of promising compounds in the early stages of the development process, minimizing the risk of late-stage failures and expediting the selection of viable drug candidates.

This study employs a comprehensive computational technique to identify and validate potential inhibitors targeting PRS in *Eimeria* species. The proposed methodology integrates: (1) virtual screening via two-stage molecular docking to identify novel inhibitors of *E. tenella* (EtPRS) from a compound library; (2) ADMET predictions to examine the drug-likeness and toxic profile of promising compounds; and (3) MD simulations to assess the binding stability and conformational dynamics of potential inhibitors with EtPRS. The combination of these computational methods is aimed to accelerate the discovery of novel and effective anticoccidial agents targeting PRS in *E. tenella*.

By advancing the understanding of drug–parasite interactions at the molecular level, this study lays a pathway for developing novel therapeutic strategies to manage the threat of coccidiosis in the poultry industry. This study also emphasizes the importance of applying combined computational approaches with experimental validation to reveal the molecular mechanisms underlying drug resistance and develop effective strategies for addressing resistance in other parasites.

## 2. Results and Discussion

### 2.1. Sequence and Model Reliability Analysis

**Figure 1:**
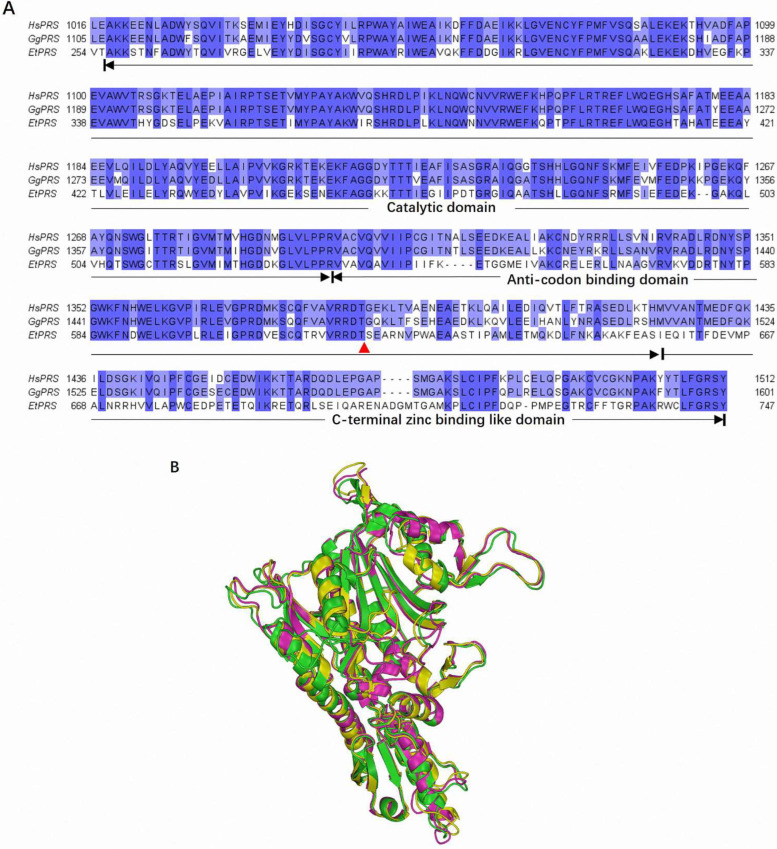
Comparative analysis of the catalytic domains in PRS enzymes across varying species and structural alignment of PRS enzymes. (**A**) Sequence alignment of the catalytic main body of PRS from *Gallus gallus* (GgPRS), *Homo sapiens* (HsPRS), and *Eimeria tenella* (EtPRS). The alignment highlights the catalytic domain (CD), anticodon binding domain (ABD), and the C-terminal zinc-binding-like domain (Z-domain). Red triangles represented the reported drug-resistant mutation sites specific to EtPRS. (**B**) Structural superimposition of EtPRS (green, PDB ID: 5xip), GgPRS (purple, modeled by AlphaFold2), and HsPRS (yellow, PDB ID: 4hvc) visualized using PyMOL. The 3D alignment reveals the spatial overlap and differences between the PRS enzymes.

The multiple sequence alignment of HsPRS, GgPRS, and EtPRS revealed significant conservation within the catalytic domain (CD), anticodon binding domain (ABD), and C-terminal zinc-binding-like domain (Z-domain). These regions are essential for enzymatic activity and structural stability, displaying key sequence similarity across the three species, as depicted in Figure 1A. Interestingly, this conservation aligns with prior studies emphasizing the evolutionary pressure to preserve critical enzymatic functions across diverse organisms [22]. The prominent conserved motifs in the CD underscores their essential role in the enzymatic activity of PRS enzymes, as highlighted in earlier research [23]. Notably, specific mutations linked to drug resistance in EtPRS were mapped onto the sequence alignment and were absent in HsPRS and GgPRS [24], suggesting potential targets for selective drug design, as discussed in previous studies [25]. The unique variations in EtPRS highlight its potential as a therapeutic target for parasitic infections, thereby providing a promising solution for drug development.

Structural validation analyses verified the high reliability of all three PRS models. Ramachandran plots detected the majority of residues within favored regions of EtPRS (85.9%), HsPRS (91.9%), and GgPRS (94.2%), with minimal outliers (Figure S1A,C,E). These findings are consistent with those previously determined in experimental structural studies [14,26]. The ERRAT scores for all models, which represent the overall quality of protein structures, were within the 86.4–96.4% range, indicating their acceptable quality (Table S1). Similarly, the ProSA-web Z-scores for all models (EtPRS: −8.92, HsPRS: −10, GgPRS: −9.87) fell within the typical range for high-resolution protein structures (Figure S1B,D,F). These results further validate the accuracy of the predicted and experimentally derived structures, aligning with previous studies by Kalman et al. [27].

Structural alignment of the three PRS enzymes demonstrated significant spatial overlap, specifically in the CD and ABDs, which are crucial for their enzymatic function (Figure 1B). Minor fluctuations were found in the loop regions and C-terminal regions, which may contribute to species-specific interactions or enzymatic efficiencies, potentially affecting enzyme–substrate interactions [28]. The predicted GgPRS structure from AlphaFold correlates closely with the experimentally determined HsPRS and EtPRS structures, with minimal variations in vital functional regions. This significant structural conservation underscores the functional similarity of PRS enzymes across species and validates the use of computational modeling for studying the PRS structure [29].

The combined sequence alignment and structural analyses emphasize the evolutionary conservation of PRS enzymes across species, specifically within key functional domains, such as the CD and ABD regions. These conserved motifs underscore their essential role in PRS activity, while unique mutations in EtPRS offer a promising option for species-specific drug targeting. Structural validation further confirmed the suitability of the PRS models for functional and comparative analyses. The AlphaFold-predicted structure of GgPRS displayed high consistency with experimentally determined structures, highlighting the accuracy of advanced computational modeling in structural prediction. However, the minor structural variations in loop regions suggest potential adaptations influencing enzyme–substrate interactions or specificity, warranting further assessment.

In short, the high-quality PRS models and the conserved sequence features identified in this study established a solid framework for future studies into PRS function, evolution, and its viability as a promising therapeutic target. The structural and functional insights revealed in this study will facilitate the practical design of selective inhibitors that target PRS in parasitic organisms, such as *E. tenella*, with minimal impact on host enzymes.

### 2.2. Docking Parameter Reliability Analysis

The molecular docking parameters and findings were thoroughly assessed to validate the accuracy and reliability of the docking simulations performed on EtPRS, HsPRS, and GgPRS. The analysis examined the grid box configurations, ligand preparation procedures, and docking outputs to ensure consistency and predictive reliability. The grid box parameters were specifically developed to include the active sites of each PRS enzyme, precisely targeting critical regions for ligand binding. As portrayed in Figure S2, the grid boxes were accurately centered and sized, with a 54–70 Å dimension range across the x-, y-, and z-axes (Table S2). These dimensions were appropriate to accommodate the docking of small molecules, such as ATP and HFG, aligning with past studies that emphasize the significant impact of precise grid box configurations in enhancing docking accuracy [30].

**Figure 2:**
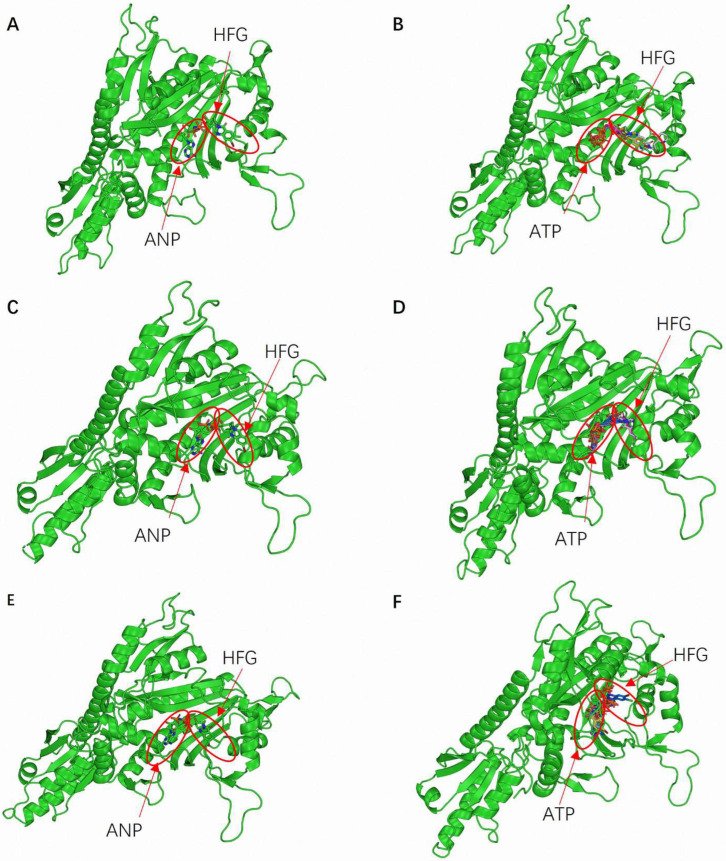
Structural and docking analysis of PRS enzyme interactions with ATP and HFG. (**A**) Binding conformations of ANP (ATP analog) and HFG in the EtPRS crystal structure (PDB ID: 5xip, chain B). (**B**) Docking results for EtPRS with ATP and HFG using iDock, QVina2, Smina, and Vina. Detailed docking parameters are provided in Table S2. (**C**) Binding conformations of ANP and HFG in the HsPRS crystal structure (PDB ID: 4hvc). (**D**) Docking results for HsPRS with ATP and HFG using the same four docking methods. (**E**) Binding conformations of ANP and HFG in the GgPRS AlphaFold model. (**F**) Docking results for GgPRS with ATP and HFG using the same four docking methods. All imaging results were visualized using PyMOL.

The selected docking parameters were validated through the accurate binding of ATP and HFG at known or predicted active sites during the simulations. This validation process confirmed the precise definition of binding sites, ensuring that the docking simulations targeted the most relevant enzyme regions. Moreover, the binding orientations of ATP and HFG were consistent across the three PRS enzymes. Figure 2A,C,E illustrates the predicted binding conformations of ATP and HFG within EtPRS, HsPRS, and GgPRS, respectively. Both ligands primarily interacted with the CD, and the complexes were stabilized by hydrogen bonds and hydrophobic interactions. These observed binding conformations were comparable to the experimental data for EtPRS and HsPRS, corroborating the accuracy of the docking procedure. Likewise, the predicted GgPRS model displayed similar binding poses, reinforcing the robustness of the proposed docking approach.

Docking simulations were performed using the four docking tools—QVina2, iDock, Smina, and Vina—to evaluate the reliability of the results further. A comparative analysis of the binding poses revealed a high consistency across the four pieces of software (Figure 2B,D,F). This inter-software agreement boosted confidence in the predicted binding modes and validated the proposed docking protocols. The alignment between experimental data and docking-based results verified the accuracy of the grid box configurations and the effectiveness of the docking methodologies. Moreover, the stereochemical integrity of the protein models was validated through energy minimization and specific validation tools, significantly enhancing the reliability of the docking simulations. Energy minimization, an essential step prior to docking, ensured that the structural models accurately represented the biologically relevant conformations, as highlighted by Pierce et al. [31].

Overall, the thorough analysis of docking parameters and binding interaction validation underscores the robustness of the docking simulations, offering a solid reference for future research into the interactions between PRS enzymes and potential therapeutic ligands. This study not only deepens the present understanding of PRS functionality but also facilitates the rational advancement of selective inhibitors targeting PRS in parasitic organisms, thereby paving the way for developing novel therapeutic strategies against parasitic infections.

### 2.3. Two-Stages Virtual Screening for EtPRS Inhibitors

**Figure 3:**
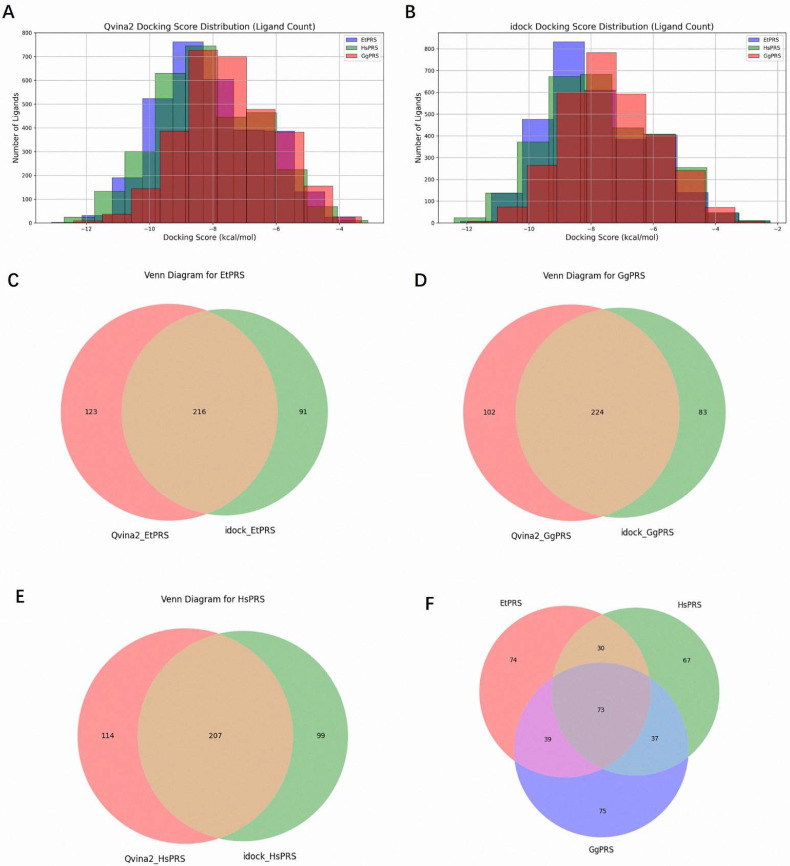
Distribution and overlap of molecular docking results for PRS enzymes with a natural compound library. Distribution of docking scores for EtPRS, HsPRS, and GgPRS interactions with a library of 3045 natural compounds using (**A**) QVina2 molecular docking and (**B**) iDock molecular docking. Color indicators: Blue = EtPRS, Green = HsPRS, and Red = GgPRS. (**C**) Venn diagram showing the top 10% docking results from QVina2 and iDock for EtPRS, revealing that both methods identified 216 compounds. (**D**) Venn diagram showing the top 10% docking results from QVina2 and iDock for GgPRS, revealing that both methods identified 224 compounds. (**E**) Venn diagram showing the top 10% docking results from QVina2 and iDock for HsPRS, revealing that both methods identified 207 compounds. (**F**) Overlap analysis of the top 10% confirmed compounds for EtPRS, GgPRS, and HsPRS from QVina2 and iDock, showing that 74 compounds are unique to EtPRS. All overlap analyses were performed using Matplotlib-venn version 1.1.1 and visualized with Matplotlib version 3.10.0.

A comprehensive virtual screening of 3045 natural compounds was performed using QVina2 and iDock to identify potential ligands for EtPRS. The top 10% of compounds with the lowest docking scores, indicative of higher binding affinity, were selected for each enzyme (EtPRS, HsPRS, and GgPRS). The distribution of docking scores is shown in Figure 3A (QVina2) and Figure 3B (iDock). Notably, compounds with lower docking scores exhibited consistent trends across both tools. Comparatively, EtPRS recorded the largest subset of high-affinity compounds, followed by GgPRS and HsPRS. This finding suggests a potential specificity for EtPRS that aligns with past studies and highlights the significance of target specificity in drug design [32].

Overlap analyses were conducted to refine the selection further. In total, 216 compounds for EtPRS were identified by both QVina2 and iDock (Figure 3C), while 224 and 207 compounds were confirmed for GgPRS and HsPRS, respectively (Figure 3D,E). Further analysis among the top compounds for all three enzymes revealed 74 compounds unique to EtPRS (Figure 3F). This step was critical in removing compounds with potential binding affinity to HsPRS or GgPRS, thereby ensuring high specificity for EtPRS. Following this rigorous process, 74 initial hits were selected for further evaluation as potential EtPRS inhibitors (Table S3).

The 74 compounds identified in stage 1 were then refined to improve the selection accuracy and ensure high specificity for EtPRS. The compounds were initially filtered according to their docking scores, with only those scoring lower than −6.5 kcal/mol in both Smina and Vina retained. This threshold, reflecting a strong binding affinity, was established in accordance with previous studies linking lower docking scores to a higher likelihood of effective binding [33]. Finally, compounds were filtered as per their specificity for EtPRS, ensuring that those with better docking scores for EtPRS compared to HsPRS and GgPRS were retained. This filtering step effectively removed non-specific inhibitors, ensuring the final selection included compounds with both strong binding affinity and high selectivity for EtPRS. Eventually, 42 high-confidence inhibitors were identified, as listed in Table S4.

From an initial library of 3045 natural compounds, the two-stage virtual screening process effectively integrated multiple docking tools (QVina2, iDock, Smina, and Vina) along with intersection and overlap analyses, resulting in a robust selection of 42 high-confidence inhibitors for EtPRS. The comprehensive methodology combined docking score thresholds, ligand pose consistency checks, and specificity filtering to identify potential inhibitors, with the final 42 compounds representing a promising pool of candidates for further experimental validation. Future studies will focus on evaluating the inhibitory activity and pharmacological potential of these compounds via in vitro and in vivo analysis, advancing the discovery and development of selective inhibitors targeting EtPRS. This systematic approach also lays a reliable foundation for the development of therapeutic strategies against parasitic infections.

### 2.4. Prediction of Pharmacokinetic Properties

The results of the ADMET analysis are shown in Table S5. Accordingly, the pharmacokinetic evaluation of the selected compounds (T5S0055, T2850, and T5574) complied favorably with Lipinski’s Rule of Five, implying their potential suitability for oral administration. These compounds demonstrated acceptable molecular weights, lipophilicity, and hydrogen bond characteristics, which are essential for efficient absorption and distribution [34]. Nevertheless, none of the compounds fulfilled the more stringent criteria set by Pfizer’s 3/75 rule or GSK’s 4/400 rule, indicating potential issues regarding oral bioavailability, necessitating further assessment in future development stages [35].

Metabolic predictions revealed that the selected compounds interacted with key cytochrome P450 (CYP) isoenzymes, including CYP2C9, CYP3A4, and CYP2D6. These interactions could substantially affect the metabolic clearance of the compounds and potentially impede drug–drug interactions, which are critical considerations during early drug development [36]. Understanding these metabolic pathways is crucial for predicting the pharmacokinetic behavior of the compounds and mitigating potential adverse effects. Notably, the compounds recorded a low likelihood of hERG channel blockage, a vital factor for evaluating cardiac safety. Additionally, the predictions indicated no mutagenic, tumorigenic, reproductive, or irritant toxicities, further supporting their overall safety profiles [37].

Although these findings underscore the potential of T5S0055, T2850, and T5574 as promising drug candidates, further structural optimization may be required to improve bioavailability and reduce metabolic liabilities. The pharmacokinetic profiles of these compounds could be enhanced for practical therapeutic applications by considering several strategies, such as modifying their chemical structures to improve solubility or mitigate CYP-mediated metabolism [38]. In summary, while the initial pharmacokinetic assessments suggest promising attributes for oral administration and safety, additional optimization and thorough in vitro and in vivo studies are crucial to characterize the pharmacokinetic profiles and therapeutic potential of these compounds. This systematic approach will facilitate the development of effective and safe inhibitors targeting EtPRS.

### 2.5. Binding Interaction and Interpretation of Selected Compounds

**Figure 4:**
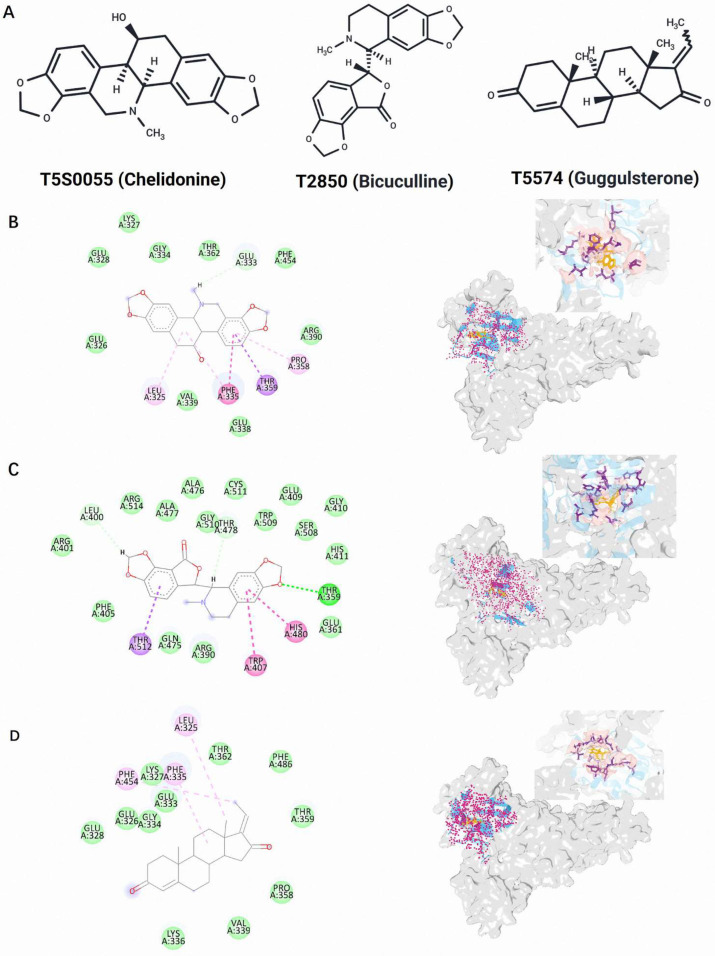
Screening of potential EtPRS inhibitors and their interactions with EtPRS. (**A**) Two-dimensional (2D) structures of the candidate EtPRS inhibitors identified in this study: T5S0055 (Chelidonine), T2850 (Bicuculline), and T5574 (Guggulsterone). Interaction conformations of EtPRS with (**B**) T5S0055, (**C**) T2850, and (**D**) T5574 based on the Vina docking results. (**Left**) Interaction analyzed using Discovery Studio Visualizer; (**Right**) Spatial interaction conformation analyzed with PyMOL.

The T5S0055 (Chelidonine), T2850 (Bicuculline), and T5574 (Guggulsterone) protein–ligand complexes were assessed to observe their binding interactions with EtPRS. The binding affinities and non-bonding interactions of the compounds were examined through 2D interaction profiles and spatial conformations, which were generated using Discovery Studio version 24.1.0.23298 and PyMOL visualization tools version 3.1.1 (Table 1; Figure 4). These analyses emphasize the compounds’ ability to effectively stabilize within the EtPRS binding site, supporting their potential as appropriate EtPRS inhibitors.

Chelidonine demonstrated an array of interactions that facilitate its strong anchoring at the active site, including pi–pi interactions with Phe335 and pi–sigma/amide interactions with Thr359. These interactions indicate a high affinity for the binding site. Furthermore, the stability of the complex was enhanced through van der Waals forces with several residues, such as Glu326, Lys327, and Arg390. Alkyl interactions with Leu325 and Pro358 also supported Chelidonine’s stable conformation within the binding pocket. The diverse interactions suggest a multifaceted binding mode that may significantly contribute to its high binding affinity and potential efficacy as an EtPRS inhibitor.

Meanwhile, Bicuculline exhibited effective binding properties through strong hydrogen bonding with Thr359, as well as pi–pi stacking interactions with Trp407 and His480. The van der Waals interactions with multiple residues, including Glu361, Arg390, and Ala476, further strengthened its binding affinity. Pi–sigma interactions with Thr512 and carbon–hydrogen bonding with Thr478 also contributed to its stability within the active site. These interactions suggest that Bicuculline possesses a dual affinity for both hydrophilic and hydrophobic residues, positioning it as a promising candidate for EtPRS inhibition [39].

Conversely, Guggulsterone depends mainly on van der Waals forces for stability, interacting with key residues, such as Glu326, Glu333, Val339, and Thr359. It also forms alkyl interactions with Phe335 and Leu325, compensating for its limited hydrogen bond formation. This reliance on hydrophobic interactions indicates that Guggulsterone may adopt a distinct binding mechanism compared to Chelidonine and Bicuculline, highlighting the essential role of non-polar interactions in stabilizing its position within the EtPRS binding site.

The findings of this study demonstrate that all three compounds form stable and diverse interactions with EtPRS, involving a mixture of hydrogen bonds, pi–pi stacking, van der Waals forces, and alkyl interactions. In comparison, Chelidonine exhibited the broadest range of interactions, which supports its strong binding affinity, while Bicuculline possessed specific interactions with both hydrophilic and hydrophobic residues, reflecting its dual affinity for the binding site. Contrarily, Guggulsterone relied primarily on non-covalent forces, particularly van der Waals and alkyl interactions, for stability. Overall, the binding profiles highlight the effective interaction between these compounds and the EtPRS active site, reinforcing their potential as appropriate inhibitors. Further experimental studies are needed to validate these interactions and optimize their binding affinities for therapeutic use.

### 2.6. Stability, Compactness, and Structural Flexibility of the EtPRS Complex

MD simulations were performed for 100 ns in triplicate on both the apo form of EtPRS and its complexes with three potential inhibitors (T5S0055, T2850, and T5574). The simulations aimed to investigate the structural stability, compactness, and flexibility of the systems, revealing key structural and conformational differences. These insights are crucial for understanding the dynamic behavior of EtPRS and its interactions with inhibitors.

**Figure 5:**
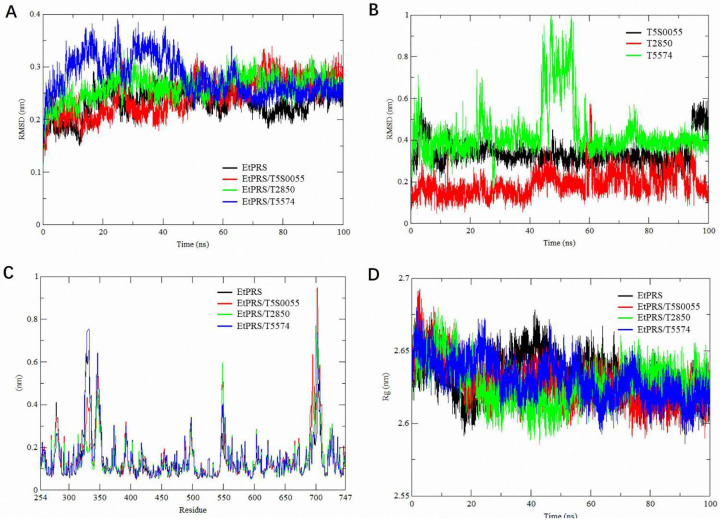
Stability analysis through 100 ns molecular dynamics simulation. (**A**) RMSD plot for the C-alpha backbone of EtPRS. The trajectories are shown for the apo form (black) and its complexes with T5S0055 (red), T2850 (green), and T5574 (blue). (**B**) Ligand RMSD plot showing trajectories for T5S0055 (black), T2850 (red), and T5574 (green). (**C**) RMSF plot for the structural backbone of EtPRS, displaying the apo form (black) and complexes with T5S0055 (red), T2850 (green), and T5574 (blue). (**D**) Radius of gyration (Rg) plot derived from a 100 ns molecular dynamics simulation. The trajectories are shown for the apo protein (black) and its complexes with T5S0055 (red), T2850 (green), and T5574 (blue). Each plot was constructed using QtGrace version 0.2.6 (https://sourceforge.net/projects/qtgrace/) (accessed on 28 December 2022).

The structural stability of the EtPRS backbone was analyzed based on the RMSD values over the simulation period. According to Figure 5A, the apo protein and the three complexes recorded average RMSD values of 0.14–0.40 nm, with all systems stabilizing within the initial 40–60 ns. In comparison, EtPRS/T5574 displayed a slightly higher RMSD during the early simulation phase, implying transient structural rearrangements that stabilized over the latter half of the trajectory. This finding aligns with recent studies indicating that ligand binding can induce conformational changes in target proteins, influencing their dynamic behavior [40,41]. The apo form of EtPRS maintained consistently lower RMSD values, suggesting stable dynamics without ligands, which corresponds with findings from similar unbound protein systems [42]. Furthermore, the ligand-specific RMSD analysis revealed distinct binding stability patterns among the three inhibitors throughout the simulation (Figure 5B). T2850 exhibited remarkably stable binding dynamics with consistently low RMSD values, while T5574 demonstrated more dynamic behavior, characterized by higher fluctuations and a distinctive peak reaching approximately 1.0 nm around 45–50 ns. Notably, T5S0055 maintained intermediate stability throughout the trajectory. These differential binding dynamics, particularly the pronounced conformational flexibility of T5574, suggest its potential for inducing significant structural adaptations in EtPRS, which may contribute to its inhibitory efficacy. These findings provide valuable insights into the molecular basis of inhibitor–target interactions and further validate T5574 as a promising EtPRS inhibitor candidate.

Structural flexibility was evaluated using RMSF values for each EtPRS backbone residue, as depicted in Figure 5C. The analysis revealed remarkably similar flexibility patterns across all systems, including both the apo form and inhibitor-bound complexes. Major fluctuations were consistently observed around residues 300–350 and near residue 700, with these regions showing comparable mobility patterns regardless of inhibitor binding. This stabilization is particularly crucial, given its enhancement of the binding affinity by mitigating the entropic penalty linked to the ligand binding [43]. The conservation of flexibility patterns across all conditions indicates that while these inhibitors effectively bind to EtPRS, they do so without dramatically altering the protein’s inherent dynamic behavior. This consistency in structural flexibility suggests that the inhibitors’ effectiveness may be more related to their specific binding interactions rather than through induced changes in protein dynamics.

The Rg was calculated to assess the system compactness during the simulations. Figure 5D shows the Rg values for the apo protein and the complexes, ranging from 2.6 to 2.65 nm. The apo form of EtPRS maintained a slightly more compact structure throughout the simulation, while EtPRS/T5574 exhibited marginal Rg variations, indicating minor conformational changes induced by its binding. This behavior suggests the ligand’s potential to regulate the protein’s structural dynamics, as previously associated with alterations in enzymatic activity [44]. In contrast, both EtPRS/T5S0055 and EtPRS/T2850 displayed stable Rg trajectories similar to the apo protein, reflecting their minimal impact on the overall protein compactness. These findings demonstrate that while both inhibitors effectively bind to EtPRS, they do so without inducing substantial changes in the protein’s overall dimensions or compactness, suggesting that their inhibitory mechanisms likely involve local interactions rather than global conformational changes.

Figure S3 illustrates the secondary structural elements of EtPRS observed throughout the simulation period. Comparatively, the apo form retained a highly stable secondary structure across the trajectory, while the inhibitor-bound systems showed subtle yet substantial changes, especially in the helical and loop regions near the binding pocket. Among the inhibitors, T5S0055 most effectively preserved the protein’s secondary structure, highlighting its stabilizing effect on the EtPRS complex. This observation is essential, as maintaining secondary structure integrity is frequently associated with the functional stability of enzymes.

Overall, the RMSD, Rg, and RMSF analyses collectively highlight that T5S0055 forms the most stable complex with EtPRS, effectively preserving its structural integrity and compactness. T2850 also displayed strong stabilizing effects, although to a slightly lesser extent, while T5574 induced mild structural variations. These findings offer valuable insights into the dynamic behavior and binding properties of EtPRS and its inhibitors, establishing a foundation for future drug development efforts. The study underscores the significance of considering structural dynamics when designing potent and selective EtPRS-targeting inhibitors, contributing to the advancement of therapeutic strategies against parasitic infections. Further experimental validation is needed to confirm the impact of these dynamic interactions in biological systems, paving the way for efficient drug design.

### 2.7. Principal Component and Free Energy Analyses of the EtPRS Complex

**Figure 6:**
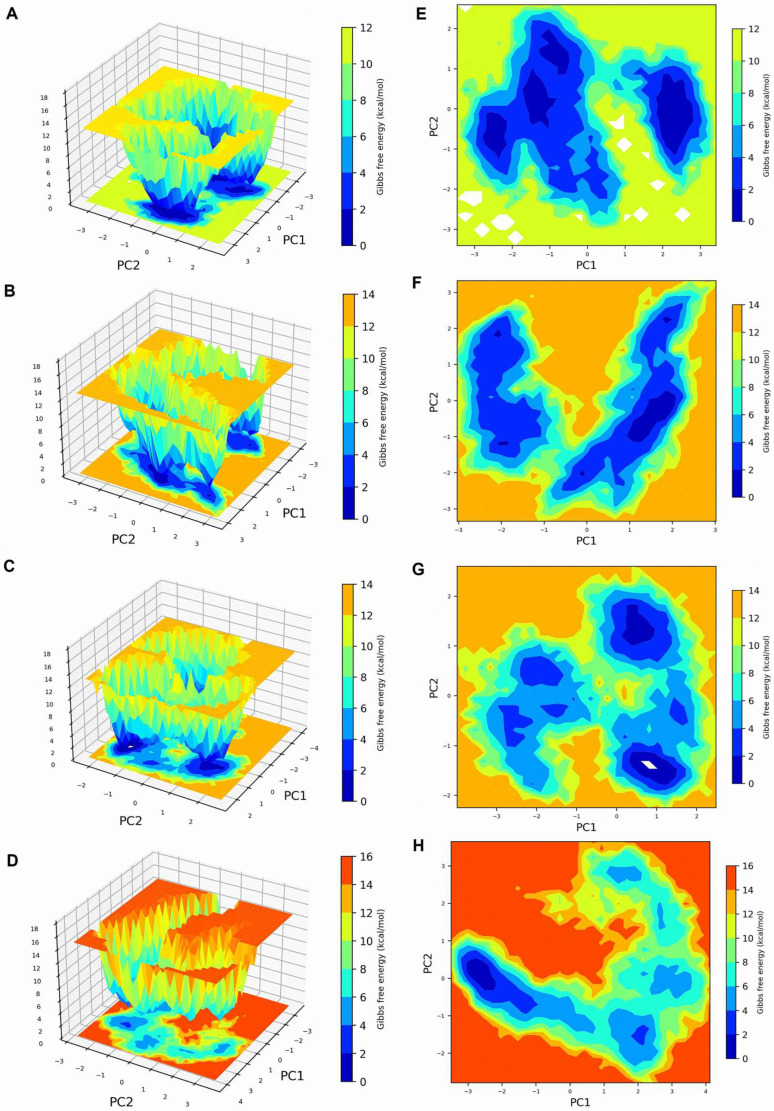
2D and 3D Free Energy Landscape (FEL) plot analysis of EtPRS with or without its inhibitor complexes. The 2D and 3D FEL plots derived from the first two principal components during 100 ns MD simulations for free (**A**,**E**) EtPRS apo protein, (**B**,**F**) EtPRS/T5S0055, (**C**,**G**) EtPRS/T2850, and (**D**,**H**) EtPRS/T5574.

PCA was conducted to assess the combined atomic motions in the apo form of EtPRS and its complexes with T5S0055, T2850, and T5574 during the 100 ns molecular dynamics simulation. The analysis emphasized eigenvectors that corresponded to the largest eigenvalues, which represented the dominant motions within the systems. Stable PCA clusters were derived from the equilibrated and stable time frames of the simulation trajectories for all four systems, as presented in Figure 6.

The computed trace values of the covariance matrices denoted the degree of motion in the systems for the EtPRS apo protein, EtPRS/T5S0055, EtPRS/T2850, and EtPRS/T5574, respectively. The apo protein recorded the smallest trace value, indicating a more compact and stable structural configuration compared to the inhibitor-bound complexes. This aligns with past studies suggesting the often reduced conformational flexibility of unbound proteins, essential for maintaining functional integrity [45]. Among the inhibitor-bound complexes, T5S0055 demonstrated a lower trace value, signifying its stabilizing effect on the protein dynamics. Conversely, T2850 and T5574 triggered more substantial collective motions, evidenced by their higher trace values, implying a more dynamic binding environment.

The system dynamics were further analyzed by generating Gibbs FEL using the first two principal components (PC1 and PC2) for all systems (Figure 6A–H). The apo protein exhibited two well-defined low-energy basins (0–12 kcal/mol), indicating two major stable conformational states (deep blue regions in Figure 6A,E). This stability indicates a well-defined conformational state frequently reported in unbound proteins [46]. Contrarily, multiple low-energy states were observed in the presence of inhibitors, suggesting conformational diversity induced by ligand binding. EtPRS/T5S0055 maintained a similar dual-basin pattern but with altered energy well shapes, suggesting moderate conformational changes (Figure 6B,F). EtPRS/T2850 displayed multiple scattered low-energy regions, indicating increased conformational flexibility (Figure 6C,G). This finding aligns with the notion that effective inhibitors induce favorable conformational changes that enhance binding affinity [47].

On the contrary, T5574 showed the most dramatic changes, with extensive high-energy regions (up to 16 kcal/mol) and a significantly altered energy landscape, implying substantial conformational rearrangements upon binding (Figure 6D,H). The observed multiple shallow energy basins suggest a higher effectiveness of T5574 in the stabilization of the protein compared to T5S0055 and T2850, potentially leading to increased binding efficacy and specificity. Such conformational state variations could affect their pharmacological profiles, as it is well-established that conformational stability can impact the binding kinetics and overall effectiveness of drug candidates [48,49].

The PCA and FEL analyses reveal marked differences in the dynamic behavior of EtPRS in its apo form and when complexed with inhibitors. Notably, T5S0055 and T2850 effectively stabilized the protein, while T5574 induced more significant conformational variations, potentially influencing their binding characteristics and efficacy. These findings align with the results from structural stability and compactness analyses, offering a comprehensive understanding of system dynamics and inhibitor interactions. These insights are valuable for guiding future drug design efforts to develop potent and selective EtPRS-targeting inhibitors, thereby advancing therapeutic strategies against parasitic infections. Additional experimental validation is essential to investigate the biological relevance of these dynamic interactions and their effects on the functional outcomes of EtPRS inhibition.

### 2.8. Binding Free Energy Analysis of the EtPRS Complexes with Potential Inhibitors

The binding affinities and energetic contributions of T5S0055, T2850, and T5574 to EtPRS were determined using the MM/GBSA method. The binding free energy was calculated based on the final 30 ns of the MD simulation trajectories, with the contributions of numerous energetic components detailed in Table 2. This analysis presents a quantitative understanding of the interactions between the inhibitors and EtPRS and their relative binding strengths, which is critical for rational drug design.

T5574 recorded the highest binding affinity, with a total binding free energy (ΔG) of −24.60 ± 2.42 kcal/mol, signifying its strong interaction with EtPRS. These results align with recent studies identifying Guggulsterone as a potent inhibitor of multiple biological targets, highlighting its potential as a lead compound for further development [50]. In comparison, T5S0055 and T2850 showed comparable binding affinities (ΔG of −14.42 ± 3.32 kcal/mol and −14.23 ± 2.51 kcal/mol, respectively). The differences in binding affinities among the inhibitors can be linked to their unique structural features and interaction profiles within the EtPRS active site.

The ΔVDWAALS interactions induced significant contributions to the binding free energy of all three inhibitors, ranging from −37.21 to −40.88 kcal/mol. These robust van der Waals interactions are critical for stabilizing the inhibitor–protein complex and have been shown to substantially influence the binding of small molecules to their targets [51]. The ΔEEL varied among the inhibitors, with T5S0055 showing moderate contributions (−11.35 ± 3.40 kcal/mol) compared to T2850 (−15.67 ± 5.51 kcal/mol) and T5574 (−5.85 ± 2.72 kcal/mol). This variation in electrostatic interactions highlights the importance of charge complementarity in ligand binding, as favorable electrostatic interactions can improve binding affinity and specificity [52].

The ΔEGB showed a positive contribution for T5S0055 (28.83 ± 3.70 kcal/mol), T2850 (32.55 ± 6.67 kcal/mol), and T5574 (18.62 ± 2.52 kcal/mol), indicating destabilizing effects. While the inhibitors interact favorably with the protein, the solvation environment may impose energetic penalties that impact the overall binding. The ΔESURF values were relatively consistent across the inhibitors, contributing minor favorable effects to binding. The balance between polar and non-polar solvation energies is vital, given its significant impact on the thermodynamic landscape of ligand binding [53].

The ΔGGAS and ΔGSOLV emerged as the key factors influencing binding affinity. T5574 demonstrated the most favorable balance between van der Waals interactions and reduced solvation penalties, resulting in its superior binding affinity. The results suggest that T5574 forms the most stable complex with EtPRS, while T5S0055 and T2850 exhibit weaker yet comparable binding. These findings offer valuable insights into the inhibitor’s binding mechanisms and provide the framework for further optimization of potential EtPRS-targeted therapeutics. The results also underscore the need for additional structural and functional studies to validate these computational predictions and assess the therapeutic potential of these inhibitors for combating parasitic infections. Future research should focus on synthesizing analogs and exploring their biological activities to boost the efficacy and selectivity of EtPRS inhibitors.

### 2.9. Study Limitations and Future Directions

While this study provides valuable insights into potential EtPRS inhibitors through molecular docking and molecular dynamics simulations, several limitations should be addressed in future research. First, our virtual screening was conducted using only the wild-type EtPRS structure, without considering known resistance mutations, particularly the L482F mutations [24] associated with halofuginone resistance. Future work should examine how these mutations impact inhibitor binding by performing comparative virtual screening against both wild-type and mutant EtPRS structures, which would help identify compounds that maintain efficacy against resistant strains.

Our analysis identified three promising compounds, Chelidonine, Bicuculline, and Guggulsterone, each exhibiting distinct binding characteristics with EtPRS. Chelidonine forms multiple hydrogen bonds and van der Waals interactions with residues such as Phe335, Glu326, and Arg390. Bicuculline demonstrates strong hydrogen bonds with Thr359 and π–π stacking interactions with Trp407 and His480. Guggulsterone, while showing the highest binding affinity through van der Waals interactions with Glu326, Val339, and Thr359, still has room for optimization.

The structural optimization of these compounds presents several opportunities for improvement. For Chelidonine, introducing additional hydrogen bond donors/acceptors and optimizing hydrophobic interactions could enhance its binding affinity. Bicuculline could benefit from enhanced π-π stacking and electrostatic interactions. Guggulsterone’s binding affinity could be further improved by introducing hydrogen bonds and optimizing hydrophobic interactions.

All three compounds face challenges with metabolic stability and bioavailability. These issues could be addressed by introducing metabolically stable groups, such as fluorine atoms or methyl groups, to reduce cytochrome P450 enzyme-mediated metabolism. Additionally, adjusting molecular properties including molecular weight, LogP values, and solubility could improve their oral bioavailability. To minimize off-target effects, structural modifications should focus on enhancing selectivity for EtPRS, particularly targeting unique residues identified in sequence alignment.

Moving forward, experimental validation through site-directed mutagenesis and binding assays will be crucial to confirm the predicted interactions. Implementation of these structural optimizations could significantly enhance the binding affinity, bioavailability, and therapeutic potential of these compounds, ultimately advancing the development of effective EtPRS-targeted anticoccidial drugs.

## 3. Materials and Methods

### 3.1. Sequence Alignment and Protein Preparation

The amino acid sequences of prolyl-tRNA synthetase (PRS) were retrieved from the National Center for Biotechnology Information (NCBI) database. The analysis included PRS sequences from *Gallus* (GgPRS, Accession number: NP_001006398.2), *Homo sapiens* (HsPRS, Accession number: NP_004437.2), and *E. tenella* (EtPRS, Accession number: CDJ42472.1). The proS_fam_I, a critical enzymatic region for PRS activity, was identified across all sequences. The sequences were aligned using Clustal Omega version 1.2.4 to examine similarities and conserved regions [54]. The alignment results were then visualized using Jalview version 2.11.4.1 [55] to identify the conserved motifs and functional regions vital to enzyme activity.

The EtPRS and HsPRS crystal structures were obtained from the RCSB Protein Data Bank, with the corresponding PDB IDs (EtPRS: 5XIP) and (HsPRS: 4HVC). Non-relevant ligands and water molecules were removed, and a single chain was retained at the beginning of each structure. Modeller version 10.5 was applied to perform local reconstruction for regions with missing loops [56]. Meanwhile, the GgPRS structure was predicted using AlphaFold version 2.3.1 [57] with default parameters. Both the predicted GgPRS model and the experimental crystal structures underwent energy minimization using Swiss Protein Data Bank Viewer (SPDBV) version 4.10 [58] to achieve optimal geometry and decrease steric clashes.

The resulting structures were assessed using the Structure Analysis and Verification Server (SAVES) version 6.0, comprising several analytical tools, such as PROCHECK [59] and ERRAT [60], to determine stereochemical quality and overall structural validity. ProSA-web (https://prosa.services.came.sbg.ac.at/prosa.php) [61] was also utilized to detect errors in the three-dimensional (3D) structures. The model with the best scores and acceptable stereochemical parameters was selected for further processing. Subsequently, protein preparation was conducted using AutoDockTools in MGLTools version 1.5.6 [62]. Water molecules, ligands, and ions were removed from the structure, followed by the addition of polar hydrogens and labeling Kollman charges to all atoms. Protonation states of histidine residues were determined at pH 7.4 using PROPKA version 3.1 [63], and the final structure was saved in PDBQT format for further docking studies.

### 3.2. Ligand Preparation

This study employed the L6010 Natural Product Library (TargetMol, version 2023.1), which constitutes 3045 compounds. Ligand preparation was performed using OpenBabel version 2.4.1 [64], where 3D conformers were generated with the command—gen3d to select the best conformer. Next, the ligands underwent energy minimization using the MMFF94 force field (—ff MMFF94—steps 5000—sd) to achieve optimal geometry. The protonation states of the ligands were adjusted to pH 7.4 (-p 7.4), followed by tautomer generation (—tautomer) to consider diverse chemical forms. Further preparation of the ligands was carried out using prepare_ligand4.py in AutoDockTools in MGLTools version 1.5.6, and the ligands were saved in PDBQT, MOL2, and SDF formats to ensure compatibility with various docking software, facilitating smoother integration into the docking workflow.

### 3.3. Preparation of Docking Parameters for Virtual Screening

Potential ligand–protein interactions were investigated by preparing docking parameters using four molecular docking tools: QuickVina version 2.1 (QVina2) [65], iDock version 1.5 [66], Smina version 1.1.2 [67], and AutoDock Vina version 1.1.2 [68]. The primary objective of the docking study was to establish reliable parameters for identifying binding sites on target proteins and evaluate the docking efficiency. Either experimentally determined crystal structures or homology models of each target protein were utilized as templates during the analysis. Furthermore, complexes of ATP and HFG, derived from experimental data or in silico models, served as essential reference points during parameter setup to identify relevant binding sites and ensure accurate docking outcomes. Grid box parameters were configured using AutoDockTools to specify regions around the active sites of target proteins, enabling docking simulations to concentrate on relevant protein regions while maintaining computational efficiency.

A test docking procedure was conducted using ATP and HFG as ligands to validate the reliability and accuracy of the docking parameters. These ligands were docked into the pre-defined grid boxes using the four docking tools, QVina2, iDock, Smina, and Vina. The docking results were compared against experimental data and predicted binding modes to evaluate the consistency and accuracy of the parameters. Only parameters that demonstrated reliable results, with precise binding orientations and robust correlations to known binding poses, were selected for subsequent virtual screening of the ligand library.

### 3.4. Two-Stage Virtual Screening for the Identification of Potential Ligands for EtPRS

#### 3.4.1. Stage 1: Initial Selection and Intersection of Docking Results

Docking simulations for EtPRS, HsPRS, and GgPRS were performed using both QVina2 and iDock. The top 10% of compounds with the lowest binding energies (highest affinity) for each protein were selected from both docking tools [69] and then compared through Venn analysis to identify the overlap of top compounds for each protein. A second Venn analysis was performed to identify compounds unique to EtPRS across all three proteins (EtPRS, HsPRS, and GgPRS). This step ensured the selection of compounds with the highest binding potential to EtPRS for further analysis. The intersections and final selections were visualized and documented using Venn diagrams.

#### 3.4.2. Stage 2: Refining the Results Based on Docking Scores and Ligand Poses

From stage 1, the identified compounds were further refined based on their docking scores and consistency in ligand poses. Only compounds with docking scores lower than −6.5 kcal/mol in both Smina and Vina, indicating strong binding affinity, were retained [33,70]. For further refinement, compounds targeting EtPRS were required to exhibit higher docking scores (in both Smina and Vina) compared to those for HsPRS and GgPRS, ensuring that the final compound selection was specific to EtPRS and demonstrated strong binding affinity and consistent poses across various docking tools.

### 3.5. Pharmacokinetic Properties Predictions

ADMETlab version 3.0 [71] was employed to predict the ADMET properties of the selected compounds, with an emphasis on critical pharmacokinetic and drug-likeness parameters. The evaluation was based on the following criteria: (1) adherence to Lipinski’s Rule of Five; (2) Pfizer’s 3/75 rule for oral bioavailability; (3) GSK’s 4/400 rule for drug-likeness; and (4) the Pan-Assay Interference Compounds (PAINS) filter to examine potential assay interference. All compounds adhered to Lipinski’s Rule of Five [72], indicating favorable drug-likeness for oral administration. Nevertheless, the compounds exhibited varying compliance with the Pfizer [73] and GSK [74] criteria, suggesting potential limitations in oral bioavailability and overall drug-likeness.

### 3.6. Molecular Dynamics Simulations

Molecular dynamics simulations were performed using GROMACS 2022 [75], with the AMBER99SB-ILDN force field for the protein. The ligands were parameterized using acpype.py, with Antechamber and ACPYPE programs in Ambertools version 23.6 [76] to define relevant ligand parameters. The simulation protocol involved several steps: primarily, each protein–ligand complex was solvated in a dodecahedral box of TIP3P water, with a minimum solute-to-box edge distance of 10 Å, and Na^+^ or Cl^−^ ions were added for neutralization. Energy minimization was conducted using the steepest descent algorithm with a convergence criterion of Fmax < 1000 kJ/mol/nm, followed by a 100 ps NVT equilibration with the V-rescale thermostat (τ = 0.1 ps) at 300 K and a 100 ps NPT equilibration with the Parrinello–Rahman barostat (τ = 2.0 ps) at 1 bar. The production MD run was carried out for 100 ns with a 2 fs time step, applying the LINCS algorithm for bond constraints.

### 3.7. Trajectory Analysis and Calculations of Binding Free Energies

Post-simulation analysis involved calculating the Root Mean Square Deviation (RMSD), Root Mean Square Fluctuation (RMSF), and radius of gyration (Rg) using GROMACS 2022 and MDAnalysis 2.0. Principal Component Analysis (PCA) was then conducted to assess the dominant collective motions of the EtPRS–inhibitor complex. Eigenvectors and eigenvalues were also calculated through essential dynamics by constructing a variance-covariance matrix using the gmx covar and gmx anaeig modules. The projections of the first two principal components were analyzed, while the Free Energy Landscape (FEL) of the EtPRS-inhibitor complex was mapped using the gmx sham module. The corresponding 3D and 2D contour plots were generated based on the variations in the principal components.

The binding free energy of the EtPRS-inhibitor complex was computed using gmx_MMPBSA version 1.6.1 [77], a specific tool for end-state free energy calculations. This method integrates molecular mechanics (MM) to assess the potential energy of molecular structures, and the solvation term is defined as the total of polar and non-polar contributions. The gmx_MMPBSA script was employed during the calculations with several key input files, including the trajectory file, topology file, and an index file that classified EtPRS and the inhibitor into distinct groups. Additional input files included GROMACS portable topology files (.itp), protein structure files (.pdb or .psf), trajectory files (.xtc), protein topology files (.gro), and run topology files (.tpr).

The binding free energy was computed by executing the final gmx_MMPBSA command on the last 10 ns of the simulation trajectory. The output provided a detailed breakdown of the energy contributions to the total energies of the receptor, ligand, and receptor–ligand complex for each frame, as well as an average value over all frames. Subsequently, the energetic contributions were grouped into GGAS (interaction energy) and GSOLV (solvation energy). GGAS was computed as the total internal (bonded) energy components (BOND, ANGLE, DIHED) and non-bonded components (VDWAALS, EEL). In contrast, GSOLV was calculated by determining polar (EGB or EPB) and non-polar (ESURF or ENPOLAR + EDISPER) contributions using Generalized Born (GB) or Poisson–Boltzmann (PB) solvation models, respectively.

### 3.8. Data Analysis and Visualization

Data analysis was carried out using Python version 3.8, utilizing specific libraries, including NumPy version 1.21.0 for numerical computations and Pandas version 1.3.0 for data manipulation and analysis. Molecular visualizations were generated using PyMOL version 2.5.0, while graphs and plots were constructed using Matplotlib version 3.4.2. Lastly, two-dimensional (2D) residual interaction diagrams were created with Discovery Studio Visualizer version 21.1.0.20298 (BIOVIA, Dassault Systèmes, San Diego, CA, USA).

## 4. Conclusions

**Figure 7:**
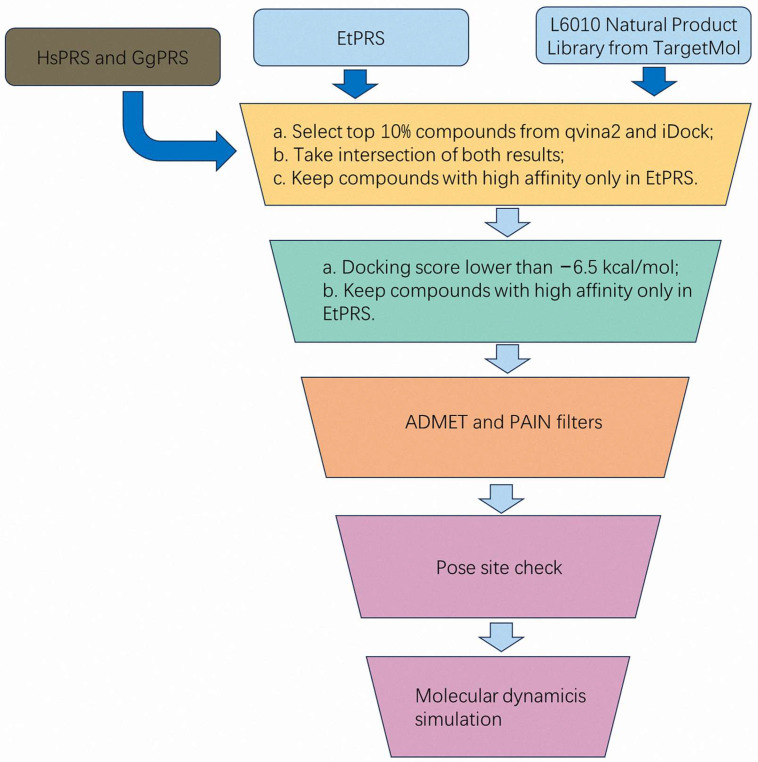
The overall methodology of this study.

This study proposed a comprehensive computational approach to identify and validate potential inhibitors targeting PRS in *E. tenella*, an essential enzyme in the coccidiosis life cycle. The methodology integrated sequence alignment, structural validation, two-stage molecular docking, ADMET predictions, and MD simulations (Figure 7). Sequence and structural analyses revealed the high conservation of PRS across species, with specific mutations in EtPRS providing promising targets for selective drug design. Rigorous validation of the docking parameter identified 42 high-confidence inhibitors from an initial library of 3045 natural compounds. Among these, Chelidonine (T5S0055), Bicuculline (T2850), and Guggulsterone (T5574) emerged as promising candidates, forming stable interactions within the EtPRS binding pocket through mixed interactions involving hydrogen bonding, van der Waals forces, and pi–pi stacking. The pharmacokinetic assessment suggested acceptable safety profiles, although considerable challenges regarding oral bioavailability and metabolic liabilities highlight the need for structural optimization. MD simulations confirmed that T5S0055 formed the most stable complex with EtPRS, preserving both structural integrity and compactness. PCA and Gibbs FEL analyses revealed distinct dynamic behaviors, with T5S0055 exhibiting the most effective stabilization effects. Binding free energy calculations identified T5574 as having the highest binding affinity, attributed to its favorably balanced van der Waals interactions and reduced solvation penalties. In summary, these findings offer valuable insights into the molecular interactions and structural dynamics of EtPRS inhibitors, establishing a strong foundation for further experimental validation and optimization of therapeutic agents. Future studies should focus on integrating computational predictions with experimental approaches to advance the development of novel anticoccidial drugs targeting PRS, ultimately improving treatment strategies against coccidiosis in the poultry industry.

## Figures and Tables

**Table 1:** Interaction results of potential inhibitors with EtPRS.

Compound	H-BOND	HBI (Pi–Pi Bond)	Van Der Waals	Carbon-Hydrogen Interaction	Pi-Sigma and Amide Interaction	Alkyl Interaction
T5S0055		PHE335	GLU326, LYS327, GLU-328, GLY334, GLU338, VAL339, THR362, ARG390, PHE454	GLU333	THR359	LEU325, PRO358
T2850	THR359	TRP407, HIS480	GLU361, ARG390, LEU400, ARG401, PHE405, GLU409, GLY410, HIS411, GLN475, ALA476, ALA477, THR478, SER508, TRP509, GLY510, CYS511, ARG514	THR478	THR512	
T5574			GLU326, LYS327, GLU328, GLU333, GLY334, LYS336, VAL339, PRO358, THR359, THR362, PHE486			PHP454, PHE335, LEU325

**Table 2:** Binding free energy (kcal/mol, average ± standard deviation) calculation of EtPRS with three potential inhibitors.

Compund Name	Compund ID	ΔVDWAALS	ΔEFL	ΔEGB	ΔESURF	ΔGGAS	ΔGSOLV	ΔTOTAL	ΔG
Chelidonine	T5S0055	−40.88 ± 2.52	−11.35 ± 3.40	28.83 ± 3.70	−4.86 ± 0.29	−52.22 ± 4.34	23.98 ± 3.73	−28.25 ± 3.32	−14.42 ± 3.32
Bicuculline	T2850	−37.21 ± 2.83	−15.67 ± 5.51	32.55 ± 6.67	−4.84 ± 0.36	−52.88 ± 7.08	27.71 ± 6.40	−25.17 ± 2.51	−14.23 ± 2.51
Guggulsterone	T5574	−38.25 ± 1.91	−5.85 ± 2.72	18.62 ± 2.52	−4.43 ± 0.14	−44.10 ± 3.24	14.20 ± 2.46	−29.90 ± 1.94	−24.60 ± 2.42

## Data Availability

The original contributions presented in this study are included in the article/Supplementary Materials. Further inquiries can be directed to the corresponding author(s).

## Supplementary Materials

The following supporting information can be downloaded at: www.mdpi.com/article/10.3390/molecules30040790/s1, Figure S1. Structural validation of PRS: (A, C, E) Ramachandran Plots and (B, D, F) PROSA Plots showing model quality. Figure S2. Grid box used for docking calculation using molecular docking. Figure S3. The secondary structural analysis of the protein during the simulation time for (A) apo protein, (B) T5S0055, (C) T2850, (D) T5574. Table S1. Structural validation results of PRS based on ramachandran plot, ERRAT, and ProSA-web. Table S2. Grid box coordinates and size parameters used used for docking. Table S3. Docking Scores of Compounds Against EtPRS, HsPRS and GgPRS Using QVina2 and iDock. Table S4. Comparison of Smina and Vina Docking Scores for Ligands Across EtPRS, HsPRS and GgPRS. Table S5 ADMET Profiles and Physicochemical Properties of Potential Inhibitor for EtPRS.

## References

[1] Sharman P.A., Smith N.C., Wallach M.G., Katrib M. (2010). Chasing the golden egg: Vaccination against poultry coccidiosis. Parasite Immunol..

[2] Allen P.C., Fetterer R.H. (2002). Recent advances in biology and immunobiology of Eimeria species and in diagnosis and control of infection with these coccidian parasites of poultry. Clin. Microbiol. Rev..

[3] Chapman H.D. (1997). Biochemical, genetic and applied aspects of drug resistance in Eimeria parasites of the fowl. Avian Pathol..

[4] Dalloul R.A., Lillehoj H.S. (2006). Poultry coccidiosis: Recent advancements in control measures and vaccine development. Expert Rev. Vaccines.

[5] Cai H., Qi N., Li J., Lv M., Lin X., Hu J., Zhang J., Liao S., Sun M. (2022). Research progress of the avian coccidiosis vaccine. Vet. Vaccine.

[6] McDonald V., Shirley M.W. (2009). Past and future: Vaccination against Eimeria. Parasitology.

[7] Mesa-Pineda C., Navarro-Ruíz J.L., López-Osorio S., Chaparro-Gutiérrez J.J., Gómez-Osorio L.M. (2021). Chicken Coccidiosis: From the Parasite Lifecycle to Control of the Disease. Front. Vet. Sci..

[8] Yan W. (2024). Feasibility Study on the Development of New Antimalarial Drugs Using Halofuginone and its Derivatives. Int. J. Public Health Med. Res..

[9] Yogavel M., Bougdour A., Mishra S., Malhotra N., Chhibber-Goel J., Bellini V., Harlos K., Laleu B., Hakimi M.A., Sharma A. (2023). Targeting prolyl-tRNA synthetase via a series of ATP-mimetics to accelerate drug discovery against toxoplasmosis. PLoS Pathog..

[10] Tegar Achsendo Y., Fransisco Candra G., Arif Maulana A., Dini K. (2024). Desain senyawa derivat halofuginon terhadap enzim Prolil-tRNA sintetase Plasmodium falciparum secara in silico. Pharmacoscript.

[11] Yu S.M., Zhao M.M., Zheng Y.Z., Zhang J.C., Liu Z.P., Tu P.F., Wang H., Wei C.Y., Zeng K.W. (2024). Chemoproteomic Strategy Identifies PfUCHL3 as the Target of Halofuginone. ChemBioChem.

[12] Ariey F., Witkowski B., Amaratunga C., Beghain J., Langlois A.C., Khim N., Kim S., Duru V., Bouchier C., Ma L. (2014). A molecular marker of artemisinin-resistant Plasmodium falciparum malaria. Nature.

[13] Pines M., Spector I. (2015). Halofuginone—The multifaceted molecule. Molecules.

[14] Zhou H., Sun L., Yang X.L., Schimmel P. (2013). ATP-directed capture of bioactive herbal-based medicine on human tRNA synthetase. Nature.

[15] Kwon N.H., Fox P.L., Kim S. (2019). Aminoacyl-tRNA synthetases as therapeutic targets. Nat. Rev. Drug Discov..

[16] Rajendran V., Kalita P., Shukla H., Kumar A., Tripathi T. (2018). Aminoacyl-tRNA synthetases: Structure, function, and drug discovery. Int. J. Biol. Macromol..

[17] Scully T.W., Jiao W., Mittelstädt G., Parker E.J. (2023). Structure, mechanism and inhibition of anthranilate phosphoribosyltransferase. Philos. Trans. R. Soc. B Biol. Sci..

[18] Sandoval D.R., Clausen T.M., Nora C., Cribbs A.P., Denardo A., Clark A.E., Garretson A.F., Coker J.K.C., Narayanan A., Majowicz S.A. (2021). The Prolyl-tRNA Synthetase Inhibitor Halofuginone Inhibits SARS-CoV-2 Infection. bioRxiv Prepr. Serv. Biol..

[19] Dias R., de Azevedo W. (2008). Molecular Docking Algorithms. Curr. Drug Targets.

[20] Alonso H., Bliznyuk A.A., Gready J.E. (2006). Combining docking and molecular dynamic simulations in drug design. Med. Res. Rev..

[21] Oli B. (2024). Revolutionizing Drug Discovery: A Comprehensive Review of Computer-Aided Drug Design Approaches. Int. J. Res. Appl. Sci. Eng. Technol..

[22] Wu H., Zeng H., Dong A., Li F., He H., Senisterra G., Seitova A., Duan S., Brown P.J., Vedadi M. (2013). Structure of the catalytic domain of EZH2 reveals conformational plasticity in cofactor and substrate binding sites and explains oncogenic mutations. PLoS ONE.

[23] Langelier M.F., Servent K.M., Rogers E.E., Pascal J.M. (2008). A third zinc-binding domain of human poly(ADP-ribose) polymerase-1 coordinates DNA-dependent enzyme activation. J. Biol. Chem..

[24] Sun P., Zhang Y., Wang C., Hu D., Liu J., Chen L., Shi F., Tang X., Hao Z., Suo J. (2023). EtcPRSMut as a molecular marker of halofuginone resistance in Eimeria tenella and Toxoplasma gondii. iScience.

[25] Cao R., Wang L., Wang H., Xia L., Erdjument-Bromage H., Tempst P., Jones R.S., Zhang Y. (2002). Role of histone H3 lysine 27 methylation in polycomb-group silencing. Science.

[26] Jain V., Yogavel M., Kikuchi H., Oshima Y., Hariguchi N., Matsumoto M., Goel P., Touquet B., Jumani R.S., Tacchini-Cottier F. (2017). Targeting Prolyl-tRNA Synthetase to Accelerate Drug Discovery against Malaria, Leishmaniasis, Toxoplasmosis, Cryptosporidiosis, and Coccidiosis. Structure.

[27] Kalman M., Ben-Tal N. (2010). Quality assessment of protein model-structures using evolutionary conservation. Bioinformatics.

[28] Burke B., Yang F., Chen F., Stehlin C., Chan B., Musier-Forsyth K. (2000). Evolutionary coadaptation of the motif 2-acceptor stem interaction in the class II prolyl-tRNA synthetase system. Biochemistry.

[29] Aulakh S.S., Bozelli J.C., Epand R.M. (2022). Exploring the AlphaFold Predicted Conformational Properties of Human Diacylglycerol Kinases. J. Phys. Chem. B.

[30] Ban T., Ohue M., Akiyama Y. (2018). Multiple grid arrangement improves ligand docking with unknown binding sites: Application to the inverse docking problem. Comput. Biol. Chem..

[31] Pierce B., Weng Z. (2008). A combination of rescoring and refinement significantly improves protein docking performance. Proteins Struct. Funct. Genet..

[32] Schenone M., Dančík V., Wagner B.K., Clemons P.A. (2013). Target identification and mechanism of action in chemical biology and drug discovery. Nat. Chem. Biol..

[33] Aydın A.D., Altınel F., Erdoğmuş H., Son Ç.D. (2021). Allergen fragrance molecules: A potential relief for COVID-19. BMC Complement. Med. Ther..

[34] Lipinski C.A. (2004). Lead- and drug-like compounds: The rule-of-five revolution. Drug Discov. Today Technol..

[35] Lagorce D., Sperandio O., Baell J.B., Miteva M.A., Villoutreix B.O. (2015). FAF-Drugs3: A web server for compound property calculation and chemical library design. Nucleic Acids Res..

[36] Zhao M., Ma J., Li M., Zhang Y., Jiang B., Zhao X., Qin S. (2021). Cytochrome P450 enzymes and drug metabolism in humans. Int. J. Mol. Sci..

[37] Mital P., Charmy K., Vivek V. (2020). An innovative impurity profiling of Avanafil using LC and LC-MS/MS with in-silico toxicity prediction. Arab. J. Chem..

[38] Di Martino R.M.C., Maxwell B.D., Pirali T. (2023). Deuterium in drug discovery: Progress, opportunities and challenges. Nat. Rev. Drug Discov..

[39] Boakye A., Okose C.Y., Heneampong I., Laryea M.K., Borquaye L.S. (2024). Molecular insights into the inhibition of Leishmania donovani O-acetylserine sulfhydrylase by cyclopropane carboxylic acid derivatives: A computational study. Discov. Chem..

[40] Döring K., Surrey T., Nollert P., Jähnig F. (1999). Effects of ligand binding on the internal dynamics of maltose-binding protein. Eur. J. Biochem..

[41] Tobi D., Bahar I. (2005). Structural changes involved in protein binding correlate with intrinsic motions of proteins in the unbound state. Proc. Natl. Acad. Sci. USA.

[42] Yang S., Kar S. (2024). Protracted molecular dynamics and secondary structure introspection to identify dual-target inhibitors of Nipah virus exerting approved small molecules repurposing. Sci. Rep..

[43] Claveria-Gimeno R., Vega S., Abian O., Velazquez-Campoy A. (2017). A look at ligand binding thermodynamics in drug discovery. Expert Opin. Drug Discov..

[44] Krishnamurthy V.M., Kaufman G.K., Urbach A.R., Gitlin I., Gudiksen K.L., Weibel D.B., Whitesides G.M. (2008). Carbonic anhydrase as a model for biophysical and physical-organic studies of proteins and protein-ligand binding. Chem. Rev..

[45] Chen S., Wiewiora R.P., Meng F., Babault N., Ma A., Yu W., Qian K., Hu H., Zou H., Wang J. (2019). The dynamic conformational landscape of the protein methyltransferase setd8. Elife.

[46] Yang L.-Q., Sang P., Tao Y., Fu Y.-X., Zhang K.-Q., Xie Y.-H., Liu S.-Q. (2014). Protein dynamics and motions in relation to their functions: Several case studies and the underlying mechanisms. J. Biomol. Struct. Dyn..

[47] Du X., Li Y., Xia Y.L., Ai S.M., Liang J., Sang P., Ji X.L., Liu S.Q. (2016). Insights into protein–ligand interactions: Mechanisms, models, and methods. Int. J. Mol. Sci..

[48] Saladino G., Gervasio F.L. (2012). New Insights in Protein Kinase Conformational Dynamics. Curr. Top. Med. Chem..

[49] Bakan A., Bahar I. (2009). The intrinsic dynamics of enzymes plays a dominant role in determining the structural changes induced upon inhibitor binding. Proc. Natl. Acad. Sci. USA.

[50] Deng R. (2007). Therapeutic effects of guggul and its constituent guggulsterone: Cardiovascular benefits. Cardiovasc. Drug Rev..

[51] Bitencourt-Ferreira G., Veit-Acosta M., de Azevedo W.F. (2019). Van der waals potential in protein complexes. Methods Mol. Biol..

[52] Verkhivker G., Agajanian S., Kassab R., Krishnan K. (2022). Probing Mechanisms of Binding and Allostery in the SARS-CoV-2 Spike Omicron Variant Complexes with the Host Receptor: Revealing Functional Roles of the Binding Hotspots in Mediating Epistatic Effects and Communication with Allosteric Pockets. Int. J. Mol. Sci..

[53] Sun H., Li Y., Tian S., Xu L., Hou T. (2014). Assessing the performance of MM/PBSA and MM/GBSA methods. 4. Accuracies of MM/PBSA and MM/GBSA methodologies evaluated by various simulation protocols using PDBbind data set. Phys. Chem. Chem. Phys..

[54] Sievers F., Higgins D.G. (2014). Clustal omega, accurate alignment of very large numbers of sequences. Methods Mol. Biol..

[55] Waterhouse A.M., Procter J.B., Martin D.M.A., Clamp M., Barton G.J. (2009). Jalview Version 2—A multiple sequence alignment editor and analysis workbench. Bioinformatics.

[56] Eswar N., Eramian D., Webb B., Shen M.Y., Sali A. (2008). Protein structure modeling with MODELLER. Methods Mol. Biol..

[57] Jumper J., Evans R., Pritzel A., Green T., Figurnov M., Ronneberger O., Tunyasuvunakool K., Bates R., Žídek A., Potapenko A. (2021). Highly accurate protein structure prediction with AlphaFold. Nature.

[58] Guex N., Peitsch M.C. (1997). SWISS-MODEL and the Swiss-PdbViewer: An environment for comparative protein modeling. Electrophoresis.

[59] Laskowski R.A., MacArthur M.W., Moss D.S., Thornton J.M. (1993). PROCHECK: A program to check the stereochemical quality of protein structures. J. Appl. Crystallogr..

[60] Colovos C., Yeates T.O. (1993). Verification of protein structures: Patterns of nonbonded atomic interactions. Protein Sci..

[61] Wiederstein M., Sippl M.J. (2007). ProSA-web: Interactive web service for the recognition of errors in three-dimensional structures of proteins. Nucleic Acids Res..

[62] Morris G.M., Huey R., Lindstrom W., Sanner M.F., Belew R.K., Goodsell D.S., Olson A.J. (2009). Autodock4 and AutoDockTools4: Automated docking with selective receptor flexiblity. J. Comput. Chem..

[63] Rostkowski M., Olsson M.H., Søndergaard C.R., Jensen J.H. (2011). Graphical analysis of pH-dependent properties of proteins predicted using PROPKA. BMC Struct. Biol..

[64] O’Boyle N., Banck M., James C., Morley C., Vandermeersch T., Hutchison G. (2011). Open Babel: An Open Chemical Toolbox. J. Cheminform..

[65] Alhossary A., Handoko S.D., Mu Y., Kwoh C.K. (2015). Fast, accurate, and reliable molecular docking with QuickVina 2. Bioinformatics.

[66] Li H., Leung K.S., Wong M.H. Idock: A multithreaded virtual screening tool for flexible ligand docking. Proceedings of the 2012 IEEE Symposium on Computational Intelligence in Bioinformatics and Computational Biology (CIBCB).

[67] Koes D.R., Baumgartner M.P., Camacho C.J. (2013). Lessons learned in empirical scoring with smina from the CSAR 2011 benchmarking exercise. J. Chem. Inf. Model..

[68] Trott O., Olson A.J. (2010). AutoDock Vina: Improving the speed and accuracy of docking with a new scoring function, efficient optimization, and multithreading. J. Comput. Chem..

[69] Feinstein W.P., Brylinski M. (2015). Calculating an optimal box size for ligand docking and virtual screening against experimental and predicted binding pockets. J. Cheminform..

[70] Mody V., Ho J., Wills S., Mawri A., Lawson L., Ebert M.C.C.J.C., Fortin G.M., Rayalam S., Taval S. (2021). Identification of 3-chymotrypsin like protease (3CLPro) inhibitors as potential anti-SARS-CoV-2 agents. Commun. Biol..

[71] Fu L., Shi S., Yi J., Wang N., He Y., Wu Z., Peng J., Deng Y., Wang W., Wu C. (2024). ADMETlab 3.0: An updated comprehensive online ADMET prediction platform enhanced with broader coverage, improved performance, API functionality and decision support. Nucleic Acids Res..

[72] Karami T.K., Hailu S., Feng S., Graham R., Gukasyan H.J. (2022). Eyes on Lipinski’s Rule of Five: A New “Rule of Thumb” for Physicochemical Design Space of Ophthalmic Drugs. J. Ocul. Pharmacol. Ther..

[73] Hughes J.D., Blagg J., Price D.A., Bailey S., DeCrescenzo G.A., Devraj R.V., Ellsworth E., Fobian Y.M., Gibbs M.E., Gilles R.W. (2008). Physiochemical drug properties associated with in vivo toxicological outcomes. Bioorganic Med. Chem. Lett..

[74] Gleeson M.P. (2008). Generation of a set of simple, interpretable ADMET rules of thumb. J. Med. Chem..

[75] Van Der Spoel D., Lindahl E., Hess B., Groenhof G., Mark A.E., Berendsen H.J.C. (2005). GROMACS: Fast, flexible, and free. J. Comput. Chem..

[76] Sousa da Silva A.W., Vranken W.F. (2012). ACPYPE—AnteChamber PYthon Parser interfacE. BMC Res. Notes.

[77] Valdés-Tresanco M.S., Valdés-Tresanco M.E., Valiente P.A., Moreno E. (2021). Gmx_MMPBSA: A New Tool to Perform End-State Free Energy Calculations with GROMACS. J. Chem. Theory Comput..

