# Supplementary material for: Structure-Based Virtual Screening of Potential Inhibitors Targeting the Prolyl-tRNA Synthetase (PRS) in Eimeria tenella: Insights from Molecular Docking, ADMET Studies, and Molecular Dynamics Simulations
Source: Molecules. 2025 Feb 8;30(4):790. doi: 10.3390/molecules30040790 (PMC11858595; doi:10.3390/molecules30040790)
Supplement: Supplementary file 1 [file molecules-30-00790-s001.zip › Supplementary file.pdf]

Supplementary files:

Structure-Based Virtual Screening of Potential Inhibitors Targeting the Prolyl-tRNA Synthetase (PRS) in *Eimeria tenella*: Insights from Molecular Docking, ADMET Studies, and Molecular Dynamics Simulations

Haiming Cai †, Shenquan Liao †, Juan Li, Minna Lv, Xuhui Lin, Yongle Song, Xiangjie Chen, Yibin Zhu, Jianfei Zhang, Nanshan Qi * and Mingfei Sun *

Key Laboratory of Livestock Disease Prevention of Guangdong Province, Key Laboratory of Avian Influenza and Other Major Poultry Diseases Prevention and Control, Ministry of Agriculture and Rural Affairs, Institute of Animal Health, Guangdong Academy of Agricultural Sciences, Guangzhou 510640, China; caihaiming@gdaas.cn (H.C.); liaoshenquan@gdaas.cn (S.L.); lijuan@gdaas.cn (J.L.); lvminna@gdaas.cn (M.L.); linxuhui@gdaas.cn (X.L.); songyongle@gdaas.cn (Y.S.); chenxiangjie@gdaas.cn (X.C.); zhuyibin@gdaas.cn (Y.Z.); zhangjfei@tom.com (J.Z.)

* Correspondence: nanshanqi@163.com (N.Q.); smf7810@126.com (M.S.)

† These authors contributed equally to this work.

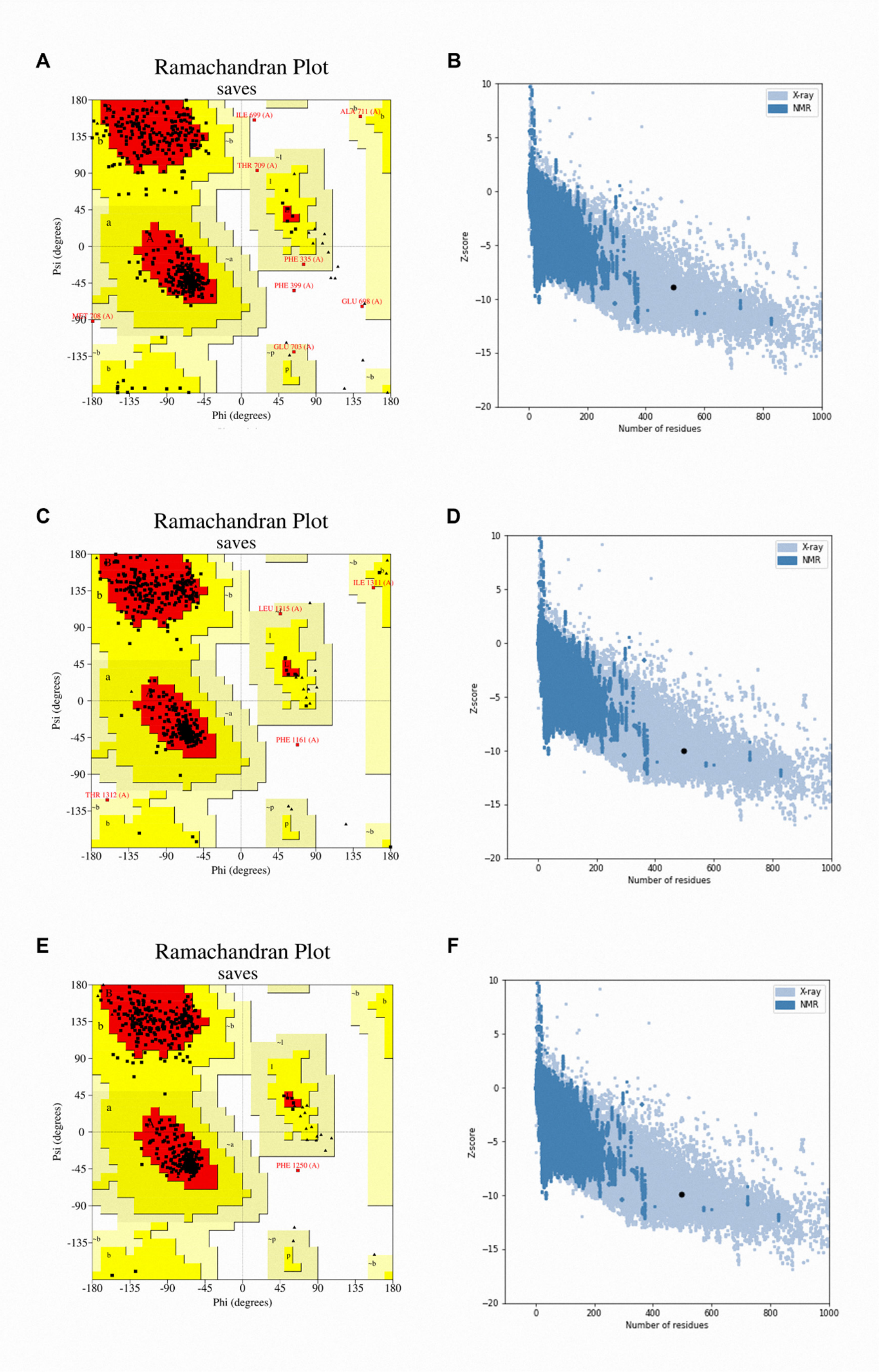

Figure S1. Structural validation of PRS: (A, C, E) Ramachandran Plots and (B, D, F) PROSA Plots showing model quality.

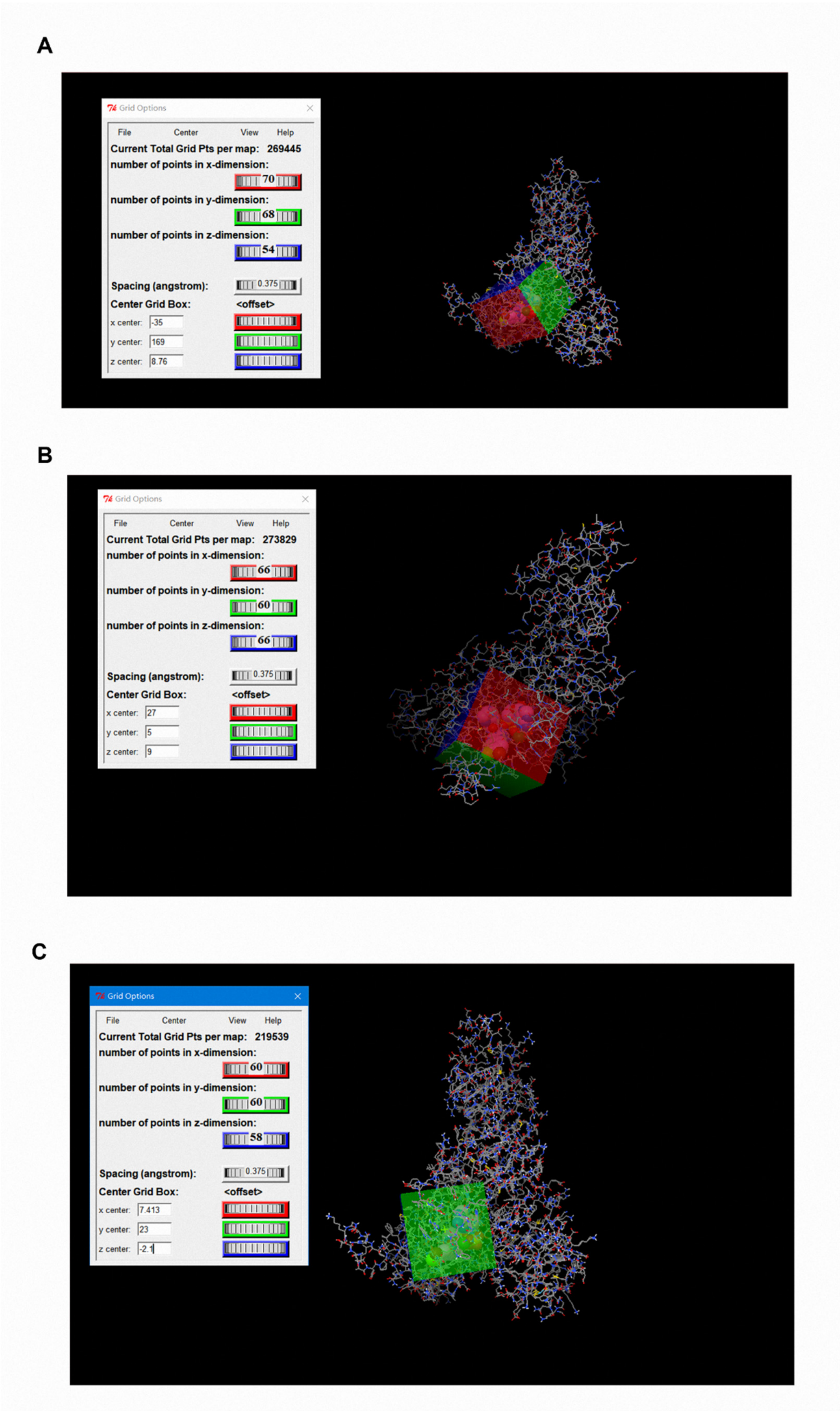

Figure S2. Grid box used for docking calculation using molecular docking.

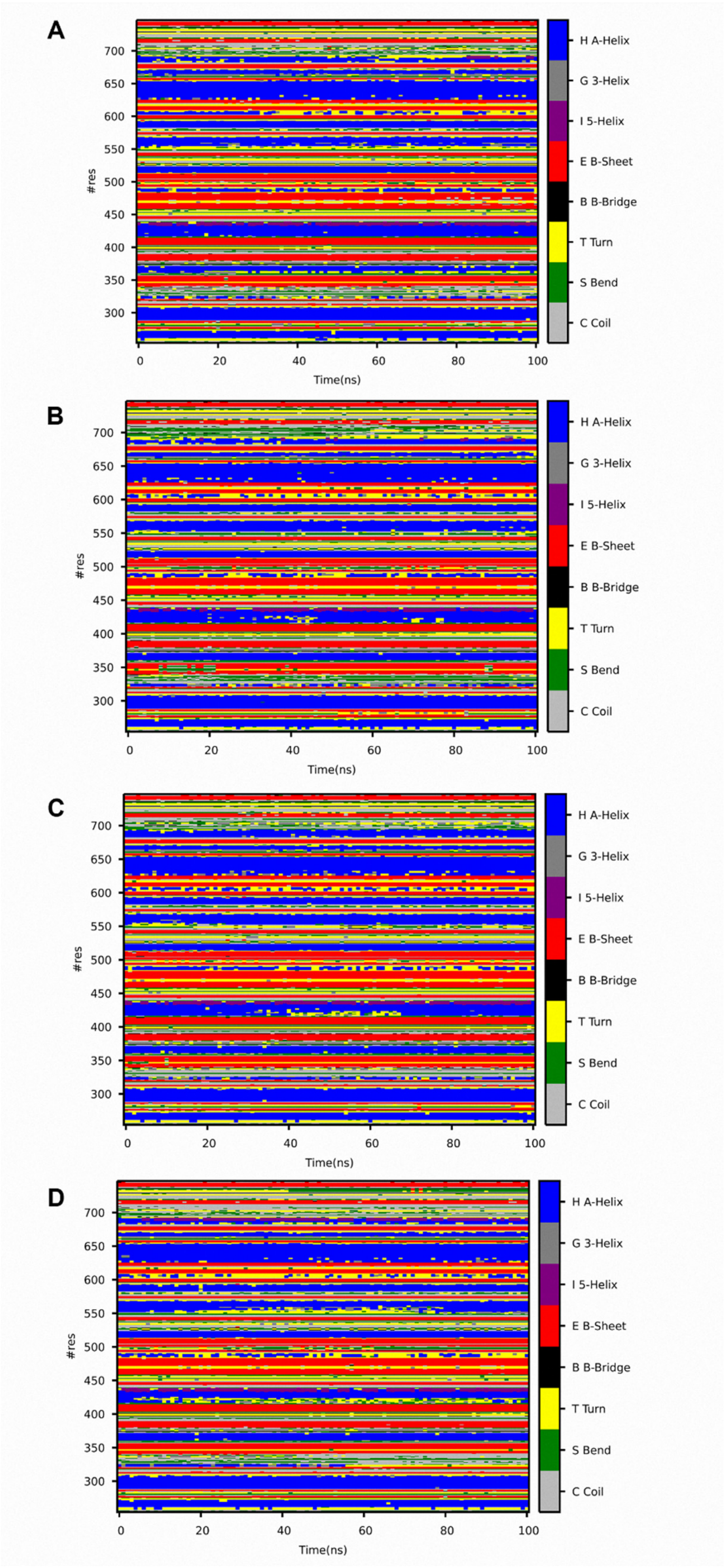

Figure S3. The secondary structural analysis of the protein during the simulation time for (A) apo protein, (B) T5S0055, (C) T2850, (D) T5574.

Table S1. Structural validation results of PRS based on ramachandran plot, ERRAT, and ProSA-web

	Ramachandran Plot Results	ERRAT overall quality factor	ProSA-web (Z-scores)
**EtPRS**	Favored Region: 85.9%<br>Allowed Region: 13.1%<br>Outlier Region: 0.9%	86.4198	-8.92
**HsPRS**	Favored Region: 91.9%<br>Allowed Region: 7.9%<br>Outlier Region: 0.2%	96.4509	-10
**GgPRS**	Favored Region: 94.2%<br>Allowed Region: 5.6%<br>Outlier Region: 0.2%	92.043	-9.87

Table S2. Grid box coordinates and size parameters used used for docking.

	Center coordinates (Å)			Size coordiantes (Å)		
	**x**	**y**	**z**	**x**	**y**	**z**
**EtPRS**	-35.000	169.000	8.760	70	68	54
**HsPRS**	27.000	5.000	9.000	66	60	66
**GgPRS**	7.413	23.000	-2.100	60	60	58
